# Supplementary material for: Importin 13-dependent axon diameter growth regulates conduction speeds along myelinated CNS axons
Source: Nat Commun. 2024 Feb 27;15:1790. doi: 10.1038/s41467-024-45908-6 (PMC10899189; doi:10.1038/s41467-024-45908-6)
Supplement: Supplementary file 1 — Supplementary Information [file 41467_2024_45908_MOESM1_ESM.pdf]

Supplemental Information

Importin 13-dependent axon diameter growth regulates conduction speeds along myelinated CNS axons

Supplementary Figures 1 and 2

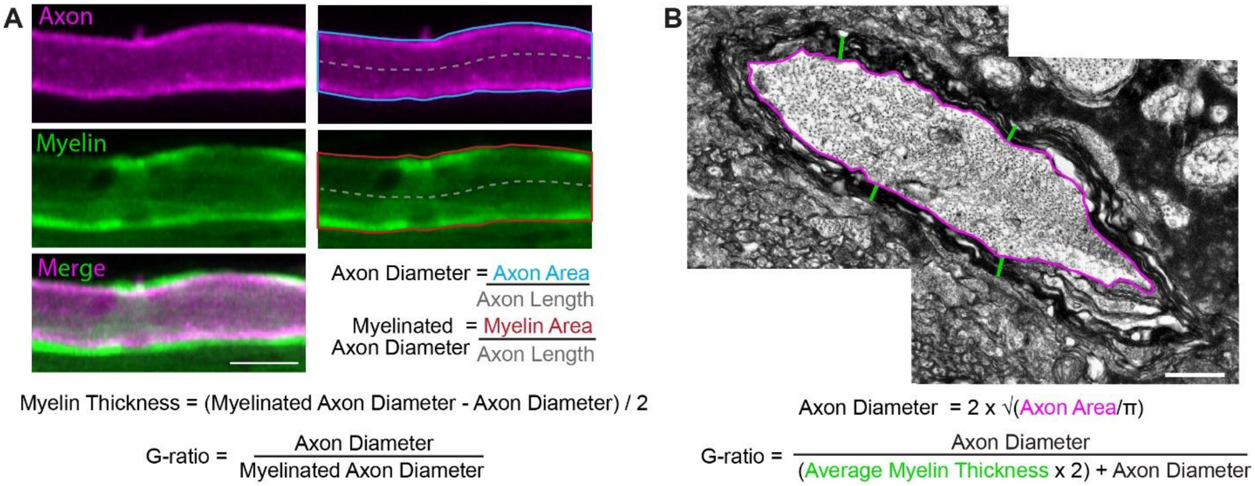

Supplementary Figure 1: Graphical description of how g-ratios were measured. (A) G-ratios measured using LSM880 AiryScan Fast images. (B) G-ratios measured using electron microscopy images. See Material and Methods for a detailed written description. Scale bars: (A) 5 µm, (B) 500 nm.

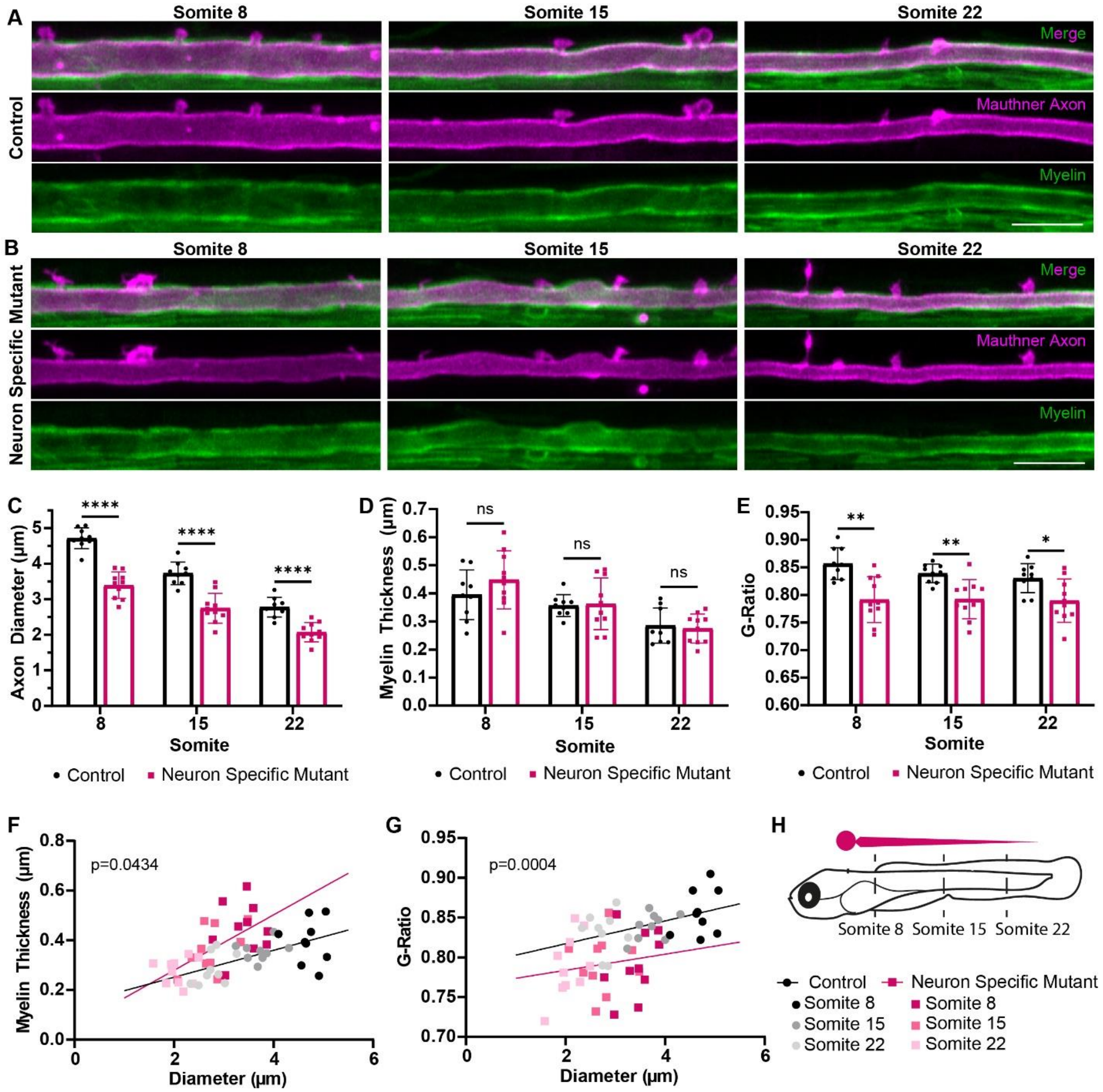

Supplementary Figure 2: Comparison of myelin thickness along the length of the Mauthner axon in neuron-specific *ipo13b* mutant animals and control siblings. (A-B) Representative images of the Mauthner axon and its myelin in 5 dpf control (A) and neuron-specific *ipo13b* mutants (B) at somite 8, 15, and 22. The Mauthner axon is labelled using Tg(hspGFF62A:Gal4); Tg(UAS:mem-Scarlet) and the myelin is labelled using Tg(mbp:eGFP-CAAX). (C-E) Quantification of axon diameter (C), myelin thickness (D) and g-ratio (E) for 5 dpf control and neuron-specific *ipo13b* mutant Mauthner axons at somite 8, 15, 22 (Two-way RM ANOVA with Tukey's multiple comparisons test, n = 9 control and 10 neuron-specific *ipo13b* mutant axons from individual fish, with repeated measures at each location. For C, p<0.0001 for somite 8, 15, and 22. For D, p=0.2455 for somite 8, p=0.8575 for somite 15, and p=0.6975 for somite 22. For E, p=0.0011 for somite 8, p=0.0026 for somite 15, and p=0.0165 for somite 22). (F) Myelin thickness plotted against axon diameter for the same control and neuron-specific *ipo13b* mutant Mauthner axons shown in C-E (The slopes of the lines are significantly different by simple linear regression test, p=0.0434). (G) G-

ratio plotted against axon diameter for the same control and neuron-specific *ipo13b* mutant Mauthner axons shown in C-E (The intercepts are significant different by simple linear regression test, p=0.0004). (H) Schematic depicting the location of somite 8, 15, and 22 along the length of the zebrafish. All data are presented as mean values +/- SD. ****p<0.0001, **p<0.01, *p<0.05. Scale bars: 10 µm. Source data are provided as a Source Data file.
