# Supplementary material for: Importin 13-dependent axon diameter growth regulates conduction speeds along myelinated CNS axons
Source: Nat Commun. 2024 Feb 27;15:1790. doi: 10.1038/s41467-024-45908-6 (PMC10899189; doi:10.1038/s41467-024-45908-6)
Supplement: Supplementary file 2 — Reporting Summary [file 41467_2024_45908_MOESM2_ESM.pdf]

nature portfolio

Corresponding author(s): David Lyons

Last updated by author(s): Jan 22, 2024

Reporting Summary

Nature Portfolio wishes to improve the reproducibility of the work that we publish. This form provides structure for consistency and transparency in reporting. For further information on Nature Portfolio policies, see our Editorial Policies and the Editorial Policy Checklist.

Statistics

For all statistical analyses, confirm that the following items are present in the figure legend, table legend, main text, or Methods section.

n/a	Confirmed	
☐	☒	The exact sample size (*n*) for each experimental group/condition, given as a discrete number and unit of measurement
☐	☒	A statement on whether measurements were taken from distinct samples or whether the same sample was measured repeatedly
☐	☒	The statistical test(s) used AND whether they are one- or two-sided<br>*Only common tests should be described solely by name; describe more complex techniques in the Methods section.*
☒	☐	A description of all covariates tested
☐	☒	A description of any assumptions or corrections, such as tests of normality and adjustment for multiple comparisons
☐	☒	A full description of the statistical parameters including central tendency (e.g. means) or other basic estimates (e.g. regression coefficient) AND variation (e.g. standard deviation) or associated estimates of uncertainty (e.g. confidence intervals)
☐	☒	For null hypothesis testing, the test statistic (e.g. *F*, *t*, *r*) with confidence intervals, effect sizes, degrees of freedom and *P* value noted<br>*Give P values as exact values whenever suitable.*
☒	☐	For Bayesian analysis, information on the choice of priors and Markov chain Monte Carlo settings
☒	☐	For hierarchical and complex designs, identification of the appropriate level for tests and full reporting of outcomes
☒	☐	Estimates of effect sizes (e.g. Cohen's *d*, Pearson's *r*), indicating how they were calculated

Our web collection on statistics for biologists contains articles on many of the points above.

Software and code

Policy information about availability of computer code

Data collection	Zen Black 2.3 (Zeiss), Ethovision XT 14 (Noldus), Clampex 10.6 (Molecular Devices)
Data analysis	Fiji Image J v1.51n, GraphPad Prism versions 8-10 (up to version 10.1.2), Adobe Photoshop 2020 (21.0.2), Adobe Illustrator 2020 (24.0.2), Ethovision XT 14, Microsoft Excel 365, Arivis Vision 4D (4.0.0), Mathematica 13.0, Custom scripts (https://github.com/skotuke/Mauthner_analysis/releases/tag/v1.0.0 and https://github.com/jasonjearly/Axon_Caliber/releases/tag/v1.0.0 doi. 10.5281/zenodo.10570003).

For manuscripts utilizing custom algorithms or software that are central to the research but not yet described in published literature, software must be made available to editors and reviewers. We strongly encourage code deposition in a community repository (e.g. GitHub). See the Nature Portfolio guidelines for submitting code & software for further information.

Data

Policy information about availability of data

All manuscripts must include a data availability statement. This statement should provide the following information, where applicable:

- Accession codes, unique identifiers, or web links for publicly available datasets
- A description of any restrictions on data availability
- For clinical datasets or third party data, please ensure that the statement adheres to our policy

All data supporting the findings of this study are available within the paper and its Supplementary Information.

Research involving human participants, their data, or biological material

Policy information about studies with human participants or human data. See also policy information about sex, gender (identity/presentation), and sexual orientation and race, ethnicity and racism.

Reporting on sex and gender	No human patients, their data, or biological material were used in this study.
Reporting on race, ethnicity, or other socially relevant groupings	No human patients, their data, or biological material were used in this study.
Population characteristics	No human patients, their data, or biological material were used in this study.
Recruitment	No human patients, their data, or biological material were used in this study.
Ethics oversight	No human patients, their data, or biological material were used in this study.

Note that full information on the approval of the study protocol must also be provided in the manuscript.

Field-specific reporting

Please select the one below that is the best fit for your research. If you are not sure, read the appropriate sections before making your selection.

☒ Life sciences ☐ Behavioural & social sciences ☐ Ecological, evolutionary & environmental sciences

For a reference copy of the document with all sections, see nature.com/documents/nr-reporting-summary-flat.pdf

Life sciences study design

All studies must disclose on these points even when the disclosure is negative.

Sample size	This was the first study to look at axon diameter is zebrafish; therefore it was not possible to do power calculations prior to the study, as the amount of diameter growth and its variability was unknown. No statistical methods were used to pre-determine sample size, but our sample sizes are similar to those reported in previous publications (Marshall-Phelps et al 2020, Xiao et al 2023, Almeida et al 2023) and was also based on including fish from multiple clutches of animals. Sample sizes for each experiments are included in the figure legends.
Data exclusions	For longitudinal time course experiments, only animals which were imaged at all time points were included.<br>For analysis of neurofilaments by electron microscopy, axons were excluded if neurofilaments could not be assessed properly due to cut angle or poor fixation. These decisions were made blind to experimental group.
Replication	All analysis were performed on animals from at least two different clutches. Axon diameter phenotype was confirmed with three different mutant alleles. All attempts at replication were successful.
Randomization	Randomization was not relevant to this study, as experimental groups were dictated by the animals genotype (control or mutant). Controls include both wild type and heterozygous siblings.
Blinding	All analyses were carried out blinded. The authors acknowledge that given the striking axon diameter phenotype of the mutant fish, genotypes were still predictable after blinding for some experimental analyses. To circumvent this, some analysis was automated (in particular, analyses of axon diameter and neurofilaments) to remove potential experimenter bias. For analyses of cell body area and volume, images were cropped to remove the axon prior to blinding and analysis.

Reporting for specific materials, systems and methods

We require information from authors about some types of materials, experimental systems and methods used in many studies. Here, indicate whether each material, system or method listed is relevant to your study. If you are not sure if a list item applies to your research, read the appropriate section before selecting a response.

Materials & experimental systems

n/a	Involved in the study
☒	☐ Antibodies
☒	☐ Eukaryotic cell lines
☒	☐ Palaeontology and archaeology
☐	☒ Animals and other organisms
☒	☐ Clinical data
☒	☐ Dual use research of concern
☒	☐ Plants

Methods

n/a	Involved in the study
☒	☐ ChIP-seq
☒	☐ Flow cytometry
☒	☐ MRI-based neuroimaging

Animals and other research organisms

Policy information about studies involving animals; ARRIVE guidelines recommended for reporting animal research, and Sex and Gender in Research

Laboratory animals	Zebrafish (danio rerio) up to 7 days post-fertilization in age. Importin 13b mutant lines: ipo13b ue57 ipo13b ue76 and ipo13b ue77. Transgenic lines used: Tg(mbp:EGFP-CAAX), Tg(hspGFF62A:Gal4) , Tg(UAS:mRFP), Tg(UAS:GFP), Tg(UAS:mem-Scarlet), Tg (U6:3sgRNA-ipo13b) and Tg(nbt:cas9).
Wild animals	No wild animals were used in this study.
Reporting on sex	Zebrafish (danio rerio) were used for experiments before the onset of sexual differentiation.
Field-collected samples	No field-collected samples were used in this study.
Ethics oversight	All experiments were performed in compliance with the UK Home Office, according to its regulations under project licenses 60/4035, 70/8436, and PP5258250.

Note that full information on the approval of the study protocol must also be provided in the manuscript.
